# Harnessing deep learning for SNP-based disease prediction in genomics

**DOI:** 10.1007/s41870-025-02624-8

**Published:** 2025-07-04

**Authors:** Colten Alme, Harun Pirim, M. Mishkatur Rahman

**Affiliations:** North Dakota State University, Fargo, ND USA

**Keywords:** Deep learning, Feature selection, SNP, Genomics, Bioinformatics

## Abstract

This study investigates the use of deep learning models to predict disease status from single nucleotide polymorphism (SNP) data. Eight GEO datasets were processed using a consistent pipeline involving genotype encoding, data cleaning, and multiple feature selection strategies. A variety of DL architectures-including feedforward networks, autoencoders, CNNs, and RNNs-were trained and evaluated. The novelty of this work lies in the standardized preprocessing, feature selection, and model training pipeline applied across all datasets, allowing for a direct and fair comparison of model performance. Results consistently showed that feedforward networks and autoencoders performed best across most datasets. This work offers a practical approach to applying deep learning in genomics with potential for future extensions.

**Supplementary Information:**

The online version contains supplementary material available at 10.1007/s41870-025-02624-8.

Introduction

Single nucleotide polymorphisms (SNPs), representing specific locations within an individual’s DNA sequence where nucleotide or base pair variations occur, are a powerful tool for disease prediction and analysis. Despite the human genome’s high degree of similarity across individuals, these single nucleotide differences can influence gene expression, potentially leading to complex diseases [1]. A visualization of an SNP can be seen in Figure 1a. The concept of Predictive Diagnostics leverages SNP analysis for early detection of disease susceptibility, enabling preventive measures and early interventions. This approach improves patient outcomes and reduces healthcare costs by preventing disease progression and avoiding expensive treatments. With the recent advancement of sequencing technologies, a large amount of SNP data has become available, which can be leveraged to build predictive models, particularly using Deep Learning techniques.

Deep Learning (DL) has been quickly emerging over the past decade as a powerful tool in the advancement of personalized medicine. This study explores the application of Deep Learning models for classifying a patient’s health status based on SNP information. Numerous DL architectures are employed across multiple diseases, with performance evaluated using accuracy, F1, specificity, and sensitivity metrics. Results indicate the potential and capacity of these models to reveal complex genotype-phenotype relationships.

## Literature review

Many researchers have been investigating the use of Deep Learning in genomics, particularly in the analysis of SNP data. This section provides a review of relevant literature on this topic.

In the study by Saeed Pirmoradi et al. [2], a self-organizing deep autoencoder was constructed to classify diseases using SNP data. The study detailed feature selection (FS) methods, addressing the challenge of high-dimensional SNP data, which often includes hundreds of thousands of SNPs. High-dimensional data poses a stiff challenge to DL applications due to the fact that many features can be irrelevant or redundant, thereby increasing training time and computational costs while reducing model accuracy [3]. Therefore, it is essential to filter out these irrelevant and redundant SNPs prior to applying DL models. Pirmoradi et al. proposed a unique FS method that achieved high accuracy scores.

To handle irrelevant features, the researchers used the Arithmetic Mean-Geometric Mean (AMGM) method, shown in Eqn. 1.

1 $$\begin{aligned} \text {AMGM Ratio} = \frac{\frac{1}{n} \sum _{i=1}^{n} x_i}{\left( \prod _{i=1}^{n} x_i \right) ^{\frac{1}{n}}} \end{aligned}$$

This approach leverages the relationship between the arithmetic mean (average) and geometric mean of features within a dataset to identify the most relevant features. Specifically, the arithmetic mean and geometric mean for each feature is calculated and put into a ratio of AM:GM. As this ratio increases, it indicates a larger disparity between these two measurements. This disparity typically signifies higher relevance or variability, as the arithmetic mean is more sensitive to variability than the geometric mean, leading to a higher ratio.

To address redundant features, the researchers applied the Cosine Redundancy Method. The Cosine Redundancy Method filters features by evaluating the cosine similarity between feature vectors [4]. When two features exhibit a high cosine similarity (near 1), it indicates that they provide nearly identical information and are thus redundant. This process ensures the creation of a diverse dataset that effectively captures the key information within the SNP data.

Pirmoradi et al. then applied a self-organizing deep autoencoder model to make predictions. This DL technique adds or removes layers to optimize predictive performance. The specific structure details are provided in the original article. This study focused on five diseases: Thyroid Cancer, Mental Retardation, Breast Cancer, Colorectal Cancer, and Autism. The accuracy results for each disease were in the 90th percentile. Performance metrics across these diseases were higher for Pirmoradi et al.’s experiment compared to other cited methods in the article [2]. In conclusion, this study demonstrates the application of a unique FS technique to a self-organizing deep autoencoder on SNP data from five different diseases, resulting in high accuracy scores.

Building on the importance of SNP feature selection, Alireza et al. [5] proposed a machine learning-based SNP feature selection framework for predicting coronary artery disease. Their model achieved strong performance with compact SNP sets, reinforcing the clinical value of optimized variant selection in genomic pipelines.

Moving from feature selection to applied disease classification, Montaez et al. [6] examined SNPs associated with Polygenic Obesity. Polygenic Obesity is influenced by genetic mutations that affect an individual’s ability to gain weight [7]. Since mutations in DNA sequence contribute to some forms of obesity, Deep Learning techniques can be applied to gain insights into obesity prediction. Montaez et al. implemented a multi-layer feed-forward artificial neural network structure, trained using stochastic gradient descent using back-propagation [6]. Performance was evaluated using metrics similar to those in the previously discussed study. This research specifically compared the performance of different-sized SNP sets, creating four models using 5, 32, 248 and 2465 SNPs. The results of this study indicated that the best-performing model used the 2465-SNP set, achieving an F-1 score of 0.4594 and an AUC (area under the curve) score of 0.9931. These metrics are promising and suggests that the number of SNPs analyzed impacts the model’s accuracy.

Demonstrating the robustness of DL techniques on large-scale non-human SNP data, Kotlarz et al. [8] employed DL to classify correct and incorrect SNP genotypes in Danish Red Dairy Cattle bulls. The study utilized two tools to analyze the genotypes of these bulls. Correct SNPs were identified when there was a full match between genotypes obtained from both tools. Conversely, Incorrect SNPs were defined as those showing mismatches between the two technologies. A deep learning architecture composed of eight sequentially connected layers was developed to classify nearly 2.3 million SNPs in the bulls. The results indicated that the model correctly identified approximately 98% of the SNPs, demonstrating an outstanding level of accuracy. Although this study has a slightly different scope than the previously discussed studies, it underscores the versatility and wide-ranging applications of DL in genomic research.

To further justify the use of deep learning over traditional models, Perelygin et al. [9] demonstrated that deep learning models outperform linear methods in capturing epistatic SNP interactions for complex diseases like obesity and type 1 diabetes. This highlights the unique capacity of DL to model non-linear genotype-phenotype relationships.

Das et al. [10] compared four traditional ML classifiers for disease prediction using symptom-based data and found Random Forest to outperform others with 99.5% accuracy. While not SNP-based, this study highlights the broader effectiveness of machine learning in clinical diagnostics. Abou El-Magd et al. [11] introduced an interpretable deep learning model for COPD diagnosis using breath data, combining CNNs with a Case-Based Reasoning system. This reflects growing interest in explainable AI within healthcare prediction tasks.

Together, these studies validate the role of deep learning in genomics and motivate our effort to evaluate models under a consistent pipeline, ensuring generalizable and reproducible results across multiple SNP datasets involving different diseases.

## Methodology

This section details the procedure involved in this study, ranging from data collection, pre-processing, feature selection, model creation and deployment, and evaluation. First, SNP datasets were obtained from publicly available sources. The data were then processed to ensure quality before being used for model training. Then, we employ numerous feature selection techniques to identify the most informative SNPs. Finally, multiple deep learning architectures were implemented for disease prediction, with performance evaluation conducted to assess their effectiveness.

### Data collection

The first step in this process is data collection. In order to create strong models, it is essential to have a sizeable and accurate SNP dataset. All SNP datasets were retrieved from the National Institutes of Health’s Gene Expression Omnibus (GEO) [12]. GEO is a public genomics data repository that holds hundreds of thousands of downloadable experiments and gene profiles, each identified by a unique GEO ID. It is important to note that in the Leukemia dataset, the Case and Control samples actually represent Acute Myeloid Leukemia (AML) and Myelodysplastic Syndrome (MDS), respectively. MDS is a less severe condition, which, in many cases, can progress into AML. The statistics and IDs of every dataset used in this study are presented in Tables 1 and 1a.

**Table 1:** Summary of case/control GEO datasets

Disease	GEO ID	# of SNPs	# of Case Samples	# of Control Samples	Experiment Citation
Autism	GSE9222	250,000	335	232	[13]
Breast Cancer	GSE16619	500,000	47	66	[14]
Colorectal Cancer	GSE34678	250,000	62	62	[15]
Mental Retardation	GSE13117	250,000	120	240	[16]
Thyroid Cancer	GSE67047	1,000,000	96	129	[17]
Leukemia	GSE47682	270,000	73 (AML)	70 (MDS)	[18]

The SNP data sets are available in genotype and phenotype formats. Genotype files include SNP data and the corresponding genotypes (AA, AB, BB, or No Call/NC), along with additional information such as DNA sequence location or allele involvement. The phenotype files contain metadata, the most crucial being the "Case or Control" designation, indicating whether a sample is affected by the disease or healthy. Both genotype and phenotype data are available as CSV files.

To prepare the data for analysis, the "Case/Control" column from the phenotype file is merged into the genotype file using Python’s Pandas library. Once the data is merged, the phenotype file is no longer needed and can be discarded. The final dataset consists of columns for each sample’s genotype at each SNP, along with a final column indicating whether the sample is a case or control.

### Data processing

The next step of the process, data processing, begins with cleaning the dataset to ensure only relevant SNPs are included. First, any SNPs where all samples share the same genotype are removed, as these do not contribute meaningful information to the model. Additionally, SNPs with more than 10% No Call (NC) values are discarded due to the excessive presence of unknown data. Once these irrelevant SNPs are removed, the next task is to address any remaining NC values. To handle this, the NC values are replaced with the most common genotype (AA, AB, or BB) for that SNP across all samples, following the method outlined by Pirmoradi et al. [2]. While this method is not perfect, it offers a good balance between accuracy and retaining relevant SNPs. Table 2a displays the SNP counts for each dataset after this initial data processing step.

After cleaning, the next challenge is encoding the genotype data, which is nominal (AA, AB, and BB), into a numerical format suitable for deep learning models. Basic encoding techniques, such as binary encoding, are inadequate because they fail to capture the meaningful differences between genotypes. For instance, assigning a 0 or 1 to each genotype ignores the inherent distinctions between AA, AB, and BB. To address this, mean encoding is used, which incorporates the relationship between the genotype and the target label (Case/Control).

In the mean encoding process, calculations are performed on a row-by-row (or SNP-by-SNP) basis. For each genotype (AA, AB, BB), the encoding value is calculated as the proportion of "Case" samples among all samples with that genotype. The mean encoding process is displayed in Eq. 2.

2 $$\begin{aligned} E(G) = \frac{\text {Number of case samples with genotype } G}{\text {Total number of samples with genotype } G} \end{aligned}$$

As seen in Eq. 2, the number of “case" targets is divided by the number of total targets for each genotype (AA, BB, or AB). For example, if a given SNP has six samples with the AA genotype and four of those samples belong to "Case" patients, then all six samples with AA are assigned an encoding value of 4/6 = 0.67. This process is repeated for each genotype across all SNPs, ensuring that the numerical representation retains meaningful biological information. Table 3a illustrates an example of the mean encoding process. Upon completion of this encoding, the data processing step is finalized.

### Feature Selection

Feature selection (FS) is a crucial preprocessing step in deep learning that enhances model performance by eliminating irrelevant and redundant features. By reducing dataset complexity, FS improves learning efficiency and mitigates overfitting. FS methods are categorized into filter, wrapper, and embedded methods. Filter methods rank features using statistical metrices, selecting the most relevant ones based on a predefined threshold. While computationally efficient, these methods do not account for interactions between features. Wrapper methods iteratively evaluate feature subsets using a predictive model. Despite their effectiveness, they are computationally expensive and can be prone to overfitting on larger datasets. Embedded methods integrate FS into model training, balancing efficiency and feature interaction analysis. Additionally there are hybrid methods, which combine two or more FS techniques. These hybrid methods have gained popularity for their ability to optimize both feature relevance and computational efficiency.

**Fig. 1:**
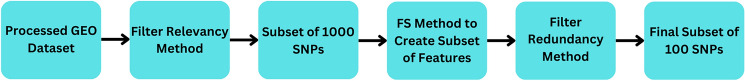
Feature selection filtering flow chart (inspired by Pirmoradi et al.)

This study initially employed the AMGM method for filtering irrelevant features, followed by Cosine Redundancy to remove redundant ones, just as in Pirmoradi et al.’s article [2]. Given that SNP datasets contain hundreds of thousands of features, this two-step process reduced the dataset to the 1000 most relevant features and then further refined it to 100 uniques features, as illustrated in Fig. 1.

To validate and compare FS techniques, several alternative methods were explored. The second most utilized FS approach combined an autoencoder with L1 regularization. An autoencoder compresses data into a lower-dimensional representation while reconstructing the input, highlighting essential features [19]. L1 (lasso) regularization further refines the selection by penalizing coefficients, removing features with near-zero contributions [20]. Another method tested was ReliefF, which evaluates feature importance based on how well features distinguish between similar samples of different classes [21]. Widely used in bioinformatics, ReliefF consides both feature relevancy and redundancy. A final approach was a hybrid technique combining Conditional Mutual Information Maximization (CMIM) with Support Vector Machine-Recursive Feature Elimination (SVM-RFE). CMIM selects features with high relevance while minimizing redundancy, while SVM-RFE iteratively removes irrelevant features using a linear SVM model, resulting in an optimized feature subset [22].

To assess the impact of feature count on model performance, experiments were conducted using subsets of 100, 1000, and 1500 features. While initial results suggested improved accuracy with more features, datasets exceeding 2500 features showed negligible performance gains and significantly higher computational costs. These findings align with Montaez et al.’s study, which demonstrated that accuracy plateaus beyond a certain subset size. This evaluation of feature selection methods ensures the identification of the most informative SNPs while maintaining computational efficiency, enabling more effective deep learning models for genomic analysis.

### Model creation

With the most relevant and unique features selected, model training was initiated. Several deep learning architectures were developed, beginning with autoencoders. The first model was a baseline autoencoder that adapted its structure to the dataset, learning an efficient feature representation [20]. This provided an initial understanding of the data’s patterns and serve as a foundation for further refinements. To improve performance, a second autoencoder was optimized using Bayesian techniques, systematically searching the hyperparameter space for the best combination of layers and activation functions [20]. Finally, a sparce autoencoder was implemented, enforcing constraints to focus on the most informative features. Each autoencoder variant was evaluated to determine the most effective representation for genomic data.

Beyond autoencoder, Recurrent Neural Networks (RNNs) were developed due to their ability to capture sequential dependencies [20]. Two architectures were explored: Bi-Directional Long Short-Term Memory (Bi-LSTM) networks and Gated Recurrent Units (GRUs). Bi-LSTMs processed input in both forward and backward directions, improving pattern recognition, while GRUs streamlined LSTM structure to reduce computational complexity [20]. Both approaches were tested to assess their potential for identifying meaningful relationships in SNP data.

Feedforward Neural Networks (FNNs), or Multi-Layer Perceptrons (MLPs), were also created. These models, consisting of fully connected layers, were optimized through hyperparameter tuning, including adjustments to layer count, neuron density, learning rate, and dropout rates. The goal was to establish a straightforward yet effective model for SNP classification.

Convolutional Neural Networks (CNNs) were explored next, leveraging convolutional layers to detect spatial relationships between SNPs. Two CNNs were developed: a standard CNN and a Residual Network (ResNet). ResNet incorporated residual connections, allowing for information to bypass layers and mitigate vanishing gradiant issues, making it well-suited for complex pattern recognition in genomic data [20].

Transformers were also tested, specifically BERT, which applies self-attention to model contextual relationships [23]. BERT’s structure, optimized for text processing, struggled to capture SNP interaction effectively, leading to suboptimal results. This highlighted the need for domain-specific adaptations before transformers could be viable for SNP classification.

Finally, stacked models were constructed to integrate the strengths of multiple architectures. Two ensemble approaches were developed: one combining a deep autoencoder with an FNN and another pairing a deep autoencoder with a Bi-LSTM network. Each component was optimized individually before being inegrated into the stacked framework. These ensemble methods aimed to enhance classification accuracy by leveraging complementary model capabilities, providing a stronger approach to SNP-based disease prediction.

### Evaluation metrics

Model performance was evaluated using four key metrics: accuracy, sensitivity, F1 score, and specificity. These metrics were chosen to provide a comprehensive understanding of prediction quality, particularly in the context of imbalanced datasets.

To ensure the evaluation was robust and generalizable, fivefold cross-validation was employed. In this method, the dataset is split into five equal parts. In each iteration, four folds are used to train the model, while the remaining fold is used for testing. This process is repeated five times, with each fold serving as the test set once. The average performance across all folds provides a reliable estimate of the model’s true effectiveness.

### Overall workflow

The final section summarizes the complete methodology employed in the study, outlining the process from data collection through model evaluation. The structured progression of each phase ensures a clear understanding of the workflow. A visual representation of this sequence is provided in Figure 2a, which outlines the fundamental steps.

The study began with the retrieval of genotypic and phenotypic data from the GEO Database from NCBI. Using R, these datasets were exported to CSV format, aligned by sample identifiers, and merged to form unified datasets that included both SNP genotypes (AA, AB, BB) and corresponding case/control labels.

Following data collection, a thorough data processing stage was conducted. SNPs with a high rate of missing values (No Call) and those showing no variability across samples were removed, as they contributed no discriminative value. Remaining missing values were accounted for by replacing them with the most common genotype for each respective SNP, ensuring dataset completeness.

Once cleaned, the genotypic data was transformed using mean encoding, a method that maps categorical genotypes to numerical values based on phenotype distributions. This step preserved biologically relevant and enhanced model interpretability.

Feature selection was then applied. Although all FS methods have been previously discussed, the order of their implementation is clarified here. The first method used was the approach introduced by Pirmoradi et al., which combined AMGM scoring with cosine similarity-based redundancy removal. All eleven models were trained and evaluated on each of the six binary case/control datasets using this FS approach. Subsequently, the same models were retrained using alternative FS strategies, including Chi-squared and mutual information ranking, to compare effectiveness and stability across selection techniques.

With a reduced and refined feature set in place, deep learning models were developed and trained. The model development phase explored a variety of architectures, beginning with a baseline autoencoder designed to adjust to the input dataset and learn an efficient representation of the underlying data structure. Building on this foundation, a second autoencoder was constructed using Bayesian optimization, which systematically searched the hyperparameter space to improve performance and generalizability. A third architecture, the sparse autoencoder, was also introduced to enforce sparsity and extract more meaningful features by limiting activation to a smaller subset of neurons. These autoencoders were applied independently to assess which architecture was most effective in representing genomic data.

To broaden the scope of model evaluation, recurrent neural networks (RNNs) were also developed due to their capacity to learn from sequential data and capture dependencies that may exist across SNP positions. These were designed and tested in parallel with the autoencoders to examine whether sequence-aware modeling could further improve prediction performance.

A key novelty of this study lies in the combination and systematic comparison of multiple FS strategies and DL architectures across several real-world, disease-specific SNP datasets. Rather than focusing on a single dataset or model type, the study was structured to test the scalability and robustness of different methods across six distinct binary case/control datasets. This comparative design enabled a more generalizable understanding of which feature selection and modeling strategies are best suited for SNP-based disease prediction and how different methods interact across datasets of varying complexity.

## Results

This section presents the results of the study. First, heat maps are presented illustrating the results of various feature selection methods and deep learning architectures. Then, the top-performing model combinations are compiled and analyzed.

### Presentation of all data

Given the extensive number of models, feature selection methods, and datasets involved, the experiments generated a large volume of results, initially organized into approximately 40 tables. To facilitate easier interpretation and comparison, these results were condensed into a series of heatmaps. These heatmaps provide a visual summary of the model performance across various conditions, including different feature selection methods (AMGM & Cosine Similarity, Autoencoder & Lasso, ReliefF, and Hybrid), varying numbers of selected SNPs (100, 1000, and 1500), and both case/control and multi-class datasets. The heatmaps are organized to allow for a straightforward comparison of model performance across these different experimental parameters. All detailed testing results are presented in the appendix (Figures 3a through 9a).

The results demonstrated high performance across most datasets. In the first iteration, using 100 SNPs selected by the AMGM & Cosine Similarity FS method, six out of eight datasets achieved several models with accuracies in the 90th percentile. The exceptions were the Colorectal Cancer and Autism datasets, which exhibited lower accuracy scores in the 50 s and 60 s. There were also instances where some models displayed metrics of 0%. This likely indicates that the testing data was dominated by a single class, leading the model to fail in correctly identifying any instances from other classes.

In the second iteration, using 100 SNPs selected by the autoencoder and Lasso FS method, there was a general decline in accuracy scores. Nonetheless, datasets such as Breast Cancer, Leukemia, Thyroid Cancer, and Smoking-Related Disease maintained high accuracy scores in the 90th percentile. Conversely, the Mental Retardation and Lymphoma datasets experienced a drop in accuracy, aligning with Autism’s improved accuracy range of 70 s and 80 s. Colorectal Cancer continued to show the lowest performance, with accuracy scores remaining around 50–60%.

In the third iteration, where the AMGM & Cosine Similarity FS method was applied with 1500 SNPs, the results were outstanding. Seven of the eight datasets achieved accuracy scores greater than 98.5% for at least one of the four models tested. However accuracy scores this high raise concerns of overfitting, indicating that the model is overly tailored to the current data and may fail to predict data from external sources. To mitigate overfitting, several precautions were implemented. Cross-validation was employed to ensure strong performance evaluation, and dropout layers were incorporated into the hyperparameters of every model. Dropout is a technique that randomly ignores neurons during model training, preventing the model from relying on any specific set of neurons [24]. These measures reduce the risk of overfitting, making the high accuracy scores more credible. Despite the overall success, the Colorectal Cancer dataset continued to produced accuracy scores in the 50 s and 60 s, prompting the exploration of alternative FS methods in subsequent iterations.

In the fourth iteration, the ReliefF Feature Selection method was employed using 1000 SNPs, yielding strong results. Five of the eight datasets achieved model accuracies of 90% or higher. The remaining three datasets-Autism, Colorectal Cancer, and Mental Retardation-also demonstrated solid performance metrics. Autism and Mental Retardation achieved accuracy metrics in the 80th percentile, while Colorectal Cancer showed a notable improvement from previous iterations, posting accuracy metrics in the 70th percentile.

In the fifth and final iteration, the previously discussed Hybrid FS method was utilized with 1000 SNPs, yielding strong results. Seven of the eight datasets saw at least one model achieving 95% accuracy or higher. However, Colorectal Cancer once again underperformed relative to the other datasets, with its top model achieving only 54% accuracy.

This presentation of data highlights the comprehensive analysis performed across various datasets and feature selection methods. The results indicate consistent performance for most datasets, with a few exceptions. The next section will provide a detailed analysis of the top-performing models, exploring their characteristics and the implications of their results.

### Top models

In this section, the top two performing models for each dataset will be presented, offering a focused and simplified view of the results. By highlighting these top-performing models, we aim to identify the most effective combinations of feature selection methods and model architectures for each dataset. To ensure robustness and generalizability, models that achieved 100% accuracy, specificity, sensitivity, and F1 score have been excluded to avoid potential overfitting. Additionally, to provide diverse methodologies, no two models from the same feature selection method are selected for the same dataset. The following table (Table 4a) summarizes the highest accuracy results achieved by the top two models for each of the eight datasets, providing a concise overview of the key findings from our extensive experimentation.

In addition to the performance results, we also provide a table detailing the hyperparameters of the top-performing models for each dataset. The hyperparameters include epochs, dropout rate, batch size, learning rate, and the number of neurons in the first and second layers (Neurons 1 and Neurons 2) for fully connected models. For other models, these parameters are replaced by the numbers of units or filter size. This information can be seen in Table 5a.

Epochs refers to the number of complete passes through the entire training dataset. The dropout rate is the fraction of neurons randomly dropped during training to prevent overfitting. Batch size represents the number of training examples used in one iteration [25]. The learning rate is the step size used at each iteration to update the model’s parameters as it moves towards minimizing the loss function. Neurons 1 and Neurons 2 denote the number of neurons in the first and second fully connected layers, respectively. Units refer to the number of units in recurrent layers, such as in RNNs. Filter size is the size of the filters in the convolutional layers of CNNs [25]. By providing these detailed hyperparameters, we offer insights into the configurations of the top-performing models, aiding the replication and further refinement of these models for future research.

It is evident from Table 4a that numerous models have demonstrated exceptional accuracy metrics. The only datasets with scores below 95% are Colorectal Cancer and Leukemia. Colorectal Cancer has consistently posed challenges throughout this study, and potential reasons for its lower performance will be explored in subsequent sections. The second highest-rated model for Leukemia is in the 80th percentile due to the exclusion of models that achieved perfect scores across all metrics. Notably, 25 combinations of deep learning architectures and feature selection methods resulted in perfect accuracy metrics for Leukemia. This phenomenon presents a dual-edged sword, warranting further discussion to assess its implications. Overall, the models have exhibited remarkable efficacy in predicting phenotypic outcomes using genotypic SNP data.

Additionally, Table 5a provides a detailed overview of the hyperparameters used in the top-performing models for each dataset. This table includes the essential parameters such as epochs, dropout rate, batch size, learning rate, and the number of neurons or filter sizes for the respective models. By examining these hyperparameters, we gain insights into the configurations that contributed to the high accuracy scores. Once again, the inclusion of this table underscores the importance of hyperparameter tuning in optimizing model performance and offers a comprehensive guide for replicating and further refining these models.

## Discussion

The following discussion aims to provide a deeper understanding of the strengths and limitations of employing deep learning for SNP genotypic data analysis, contributing valuable insights to the ongoing development of precision medicine. As briefly mentioned earlier, focus will initially be placed on the challenges associated with the Colorectal Cancer dataset. Several factors may explain why this dataset is not yielding accuracy scores comparable to the others. Firstly, Colorectal Cancer is a complex and heterogeneous disease, involving intricate genetic interactions that standard feature selection methods and deep learning architectures may not fully capture. This heterogeneity implies the presence of multiple subtypes, each with distinct characteristics, complicating the model’s ability to identify consistent patterns within the data. Additionally, the SNP data used may not be the most relevant or informative for this particular disease, potentially contributing to lower predictive performance. The presence of noise in the data can further impede the model’s accuracy. Lastly, although preprocessing methods were rigorously applied and repeated to ensure accuracy, suboptimal preprocessing specific to this dataset cannot be entirely ruled out. However, this is considered less likely, as other datasets subjected to the same preprocessing steps yielded high results. Overall, the combination of disease complexity, potential data irrelevance, and noise likely contributes to the lower accuracy scores observed for the Colorectal Cancer dataset, necessitating further investigation and potentially more sophisticated approaches.

Pivoting to Leukemia, we observe the other end of the spectrum, where this dataset exhibited remarkable accuracy metrics, with 25 model combinations achieving perfect scores. Several potential explanations for these high metrics. On the positive side, Leukemia may have well-defined genetic markers (SNPs) that are highly informative and conducive to accurate predictions. Additionally, the dataset could be of exceptionally high quality, with minimal noise and highly relevant SNPs selected, facilitating effective learning by the models. Furthermore, the homogeneity of the disease might present less genetic and phenotypic variability, enabling the models to identify consistent patterns more easily.

However, these high scores could also have negative explanations. Overfitting is a significant concern, as models might perform exceptionally well on the training data but fail to generalize to new, unseen data. This concern is highlighted by the perfect scores achieved by multiple model combinations. Also, data leakage, where information from the test set inadvertently influences the training process, could result in artificially inflated performance metrics. Furthermore, redundant features in the dataset might enhance the model’s performance without genuinely contributing to its predictive power.

Overall, while the high accuracy metrics for Leukemia are promising, it is crucial to scrutinize these results to ensure they reflect genuine predictive power rather than artifacts of the data or modeling process. Understanding the underlying reasons for these exceptional metrics will enable a better assessment of the models’ robustness and reliability, ultimately helping to improve predictive modeling in genomics.

Similar models were developed in previous studies. Pirmoradi et al. employed a deep autoencoder and the AMGM feature selection method on five of the same datasets used in this study: Autism, Breast Cancer, Colorectal Cancer, Mental Retardation, and Thyroid Cancer. Their results showed that each dataset achieved accuracy metrics higher than 94%, including Colorectal Cancer. Additionally, two of their datasets, Breast Cancer and Thyroid Cancer, achieved perfect accuracy scores [2].

This remarkable success, similar to our study, raises concerns about overfitting. Upon reviewing their methodology, it appears that minimal precautions were taken to mitigate this issue. For example, there were no dropout layers incorporated into the autoencoder structure, and their testing lacked cross-validation. Such omissions could lead to an overrepresentation of the majority class, thereby inflating the accuracy metrics. So, despite the high accuracy scores reported in their study, it is essential to critically assess the reasons for these metrics.

The six remaining datasets-Autism, Breast Cancer, Lymphoma, Mental Retardation, Smoking-Related Disease, and Thyroid Cancer-achieved impressive accuracy metrics, hovering near 98% and 99%. These results reflect the effectiveness of the deep learning models and feature selection techniques employed in this study. Several factors contribute to these strong outcomes, each with both positive and potentially negative implications that warrant further examination.

The primary reason for these strong scores is the effective feature selection and robust deep learning architectures used. The selected feature selection methods were highly effective in identifying the most relevant and informative SNPs for each disease, ensuring the models were well-suited for genomic data analysis. Plus, these results reflect the overall quality of the data used, as high-quality data with minimal noise and relevant features contribute to models achieving high accuracy metrics. Lastly, high accuracies could indicate that the diseases represented in the data have genetic markers that facilitate easy identification and predictions by the models.

While high accuracy scores are promising, they also pose a risk of overfitting. This issue has been consistently addressed throughout this article, with precautions such as cross-validation and regularization techniques employed to mitigate it. Another reason for caution with high scores is the potential impact of imbalanced data. For example, a dataset with significantly more controls than cases might bias the model towards the majority class, leading to accuracy metrics that do not accurately reflect the model’s performance on the minority class. However, the datasets within this study are generally balanced, minimizing this concern. Although there is some imbalance within the multi-class datasets, it is not extreme and should not significantly affect the results.

Data leakage is another critical issue to be vigilant about. Data leakage occurs when information from the test set inadvertently influences the training process, leading to artificially high performance metrics that, like overfitting, do not generalize to external datasets. To counter this, strict separation of training and test data, along with careful validation procedures, were implemented. Also, high accuracy within the datasets does not necessarily imply that the models will perform equally well on independent datasets. The models may have learned specific patterns or relationships unique to the datasets utilized in this study, limiting their applicability to broader populations. These concerns must be considered when evaluating the high performance of these deep learning models.

The high accuracy metrics observed in these eight datasets are encouraging, highlighting the potential of DL models in genomics. However, it is paramount to critically evaluate these results to ensure they are not products of overfitting, imbalanced classes, or data leakage. Future research should focus on validating these models on independent datasets and exploring ways to neutralize such biases. By addressing these concerns, we can enhance the reliability of predictive models in genomics research, ultimately contributing to the advancement of precision medicine.

## Conclusion

In this study, numerous deep learning architectures and feature selection techniques were applied to eight disease SNP datasets gathered from GEO. Accuracy metrics (accuracy, F-1 score, specificity, and sensitivity) were gathered using cross-validation. Results were strong, with several models achieving high accuracy metrics across most datasets. Notably, the deep learning models demonstrated robust performance in predicting disease outcomes based on SNP data.

Results were strong, as the top-performing models exhibited high accuracy. Several datasets even achieved perfect or near-perfect scores, underscoring the potential of deep learning approaches in genomic applications. However, the Colorectal Cancer dataset posed challenges, scoring much lower than the rest. This discrepancy highlights the complexity of this particular disease.

This study’s findings are significant in the context of genomics. The ability of deep learning models to uncover intricate genotype-phenotype relationships and predict disease presence demonstrates their potential to revolutionize personalized medicine. By leveraging SNP data, these models provide valuable potential insights into the genetic basis of diseases, paving the way for more targeted and effective solutions.

Although the results were promising, several challenges and limitations were encountered. Overfitting was a major concern, especially when datasets achieved perfect accuracy scores. While cross-validation, excluding perfect scores from the top models table (Table 4a), and implementing dropout layers were employed to mitigate this issue, further measures may be necessary. Additionally, concerns such as class imbalance, data leakage, and lack of generalizability were also investigated.

Future research should focus on validating these models using independent datasets to ensure their applicability and reliability in real-world scenarios. By "independent datasets", we refer to data that was not utilized during training or validation, including datasets from alternative sources, populations, or varying experimental conditions. Such validation is crucial for assessing the generalizability of these models and mitigating the risk of overfitting, ensuring that they perform well beyond the datasets they were originally trained on. Additionally, cross-validation with external datasets from multiple institutions or geographic regions could provide deeper insight into model strength and reproducibility.

Beyond validation, exploring novel feature selection methods and advanced deep learning architectures could further enhance model performance. Investigating hybrid approaches that integrate graph-based learning with traditional machine learning techniques may offer improved capabilities. Furthermore, expanding the scope of datasets to include more diverse populations would enhance model fairness and applicability across different health care settings.

Another promising direction is the use of transfer learning and self-supervised learning to improve model adaptability. Pre-trained models on large biomedical datasets could reduce data requirements while maintaining high accuracy. Real-time adaptation, where models update with new patient data could further enhance relevance. Also, collaborating with clinicians and other medical professionals to develop user-friendly decision-support systems would help with the integration of these models into real-world medical applications, improving patient outcomes. These advancements, coupled with rigorous validation and clinical integration, will ultimately pave the way for the realization of deep learning’s full potential in genomics and personalized medicine, transforming healthcare practices.

## Supplementary Information

Below is the link to the electronic supplementary material.

Supplementary file 1 (pdf 6111 KB)

## Data Availability

All working codes, data, and documents related to this study can be found at https://github.com/CEL-lab/SNP_Deep-Learning. Supplementary tables and figures can be found at https://figshare.com/s/53bb42e76a23b1e91930.
