# Supplementary material for: Harnessing deep learning for SNP-based disease prediction in genomics
Source: Int J Inf Technol. 2025 Jul 4;17(7):3791–800. Online ahead of print. doi: 10.1007/s41870-025-02624-8 (PMC12356146; doi:10.1007/s41870-025-02624-8)
Supplement: Supplementary file 1 — (pdf 6111 KB) [file 41870_2025_2624_MOESM1_ESM.pdf]

1 Supplementary Figures and Tables

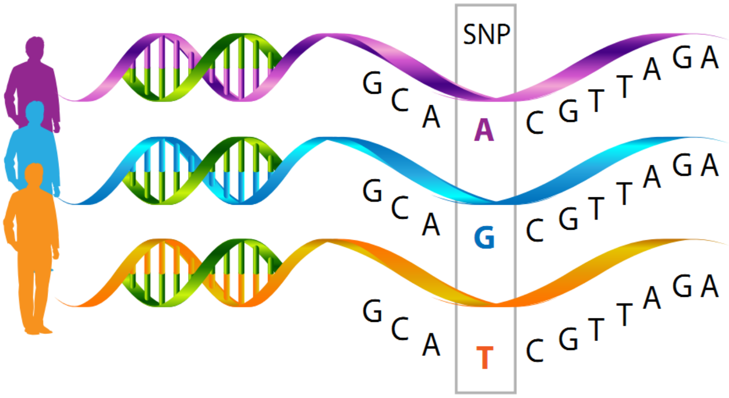

Figure 1a: Illustration of an SNP

Table 1a: Summary of Multi-Class GEO Datasets

Disease	GEO ID	# of SNPs	# of First Class	# of Second Class	# of Third Class	Experiment Citation
Small Airway Disease	GSE44865	440,000	37 (Healthy Non-Smokers)	70 (Healthy Smokers)	10 (Non-Healthy Smokers)	[16]
Lymphoma	GSE57612	240,000	79 (Type GCB Lymphoma)	49 (Type ABC Lymphoma)	20 (Unclassified / Type 3 Lymphoma)	[17]

Table 2a: SNP Counts Before & After Processing

Disease	SNP Count Pre-Processing	SNP Count Post-Processing
Autism	250,000	220,000
Breast Cancer	500,000	460,000
Colorectal Cancer	300,000	250,000
Mental Retardation	250,000	190,000
Thyroid Cancer	1,000,000	790,000
Leukemia	270,000	190,000
Small Airway Disease	440,000	430,000
Lymphoma	240,000	200,000

Table 3a: Example of Mean Encoding Process

Sample	Genotype	Label	Mean Encoding Value
1	AA	Case	3 (# of Case) / 5 (# of Instances) = 0.6
2	AA	Case	3/5 = 0.6
3	AA	Control	3/5 = 0.6
4	AA	Case	3/5 = 0.6
5	AA	Control	3/5 = 0.6
6	AB	Case	1/2 = 0.5
7	AB	Control	1/2 = 0.5
8	BB	Control	4/6 = 0.67
9	BB	Case	4/6 = 0.67
10	BB	Case	4/6 = 0.67
11	BB	Control	4/6 = 0.67
12	BB	Case	4/6 = 0.67
13	BB	Case	4/6 = 0.67

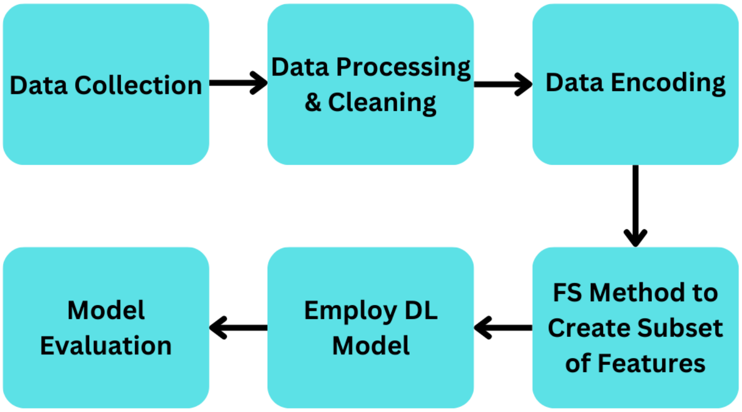

Figure 2a: Basic Flow Chart of Research Process

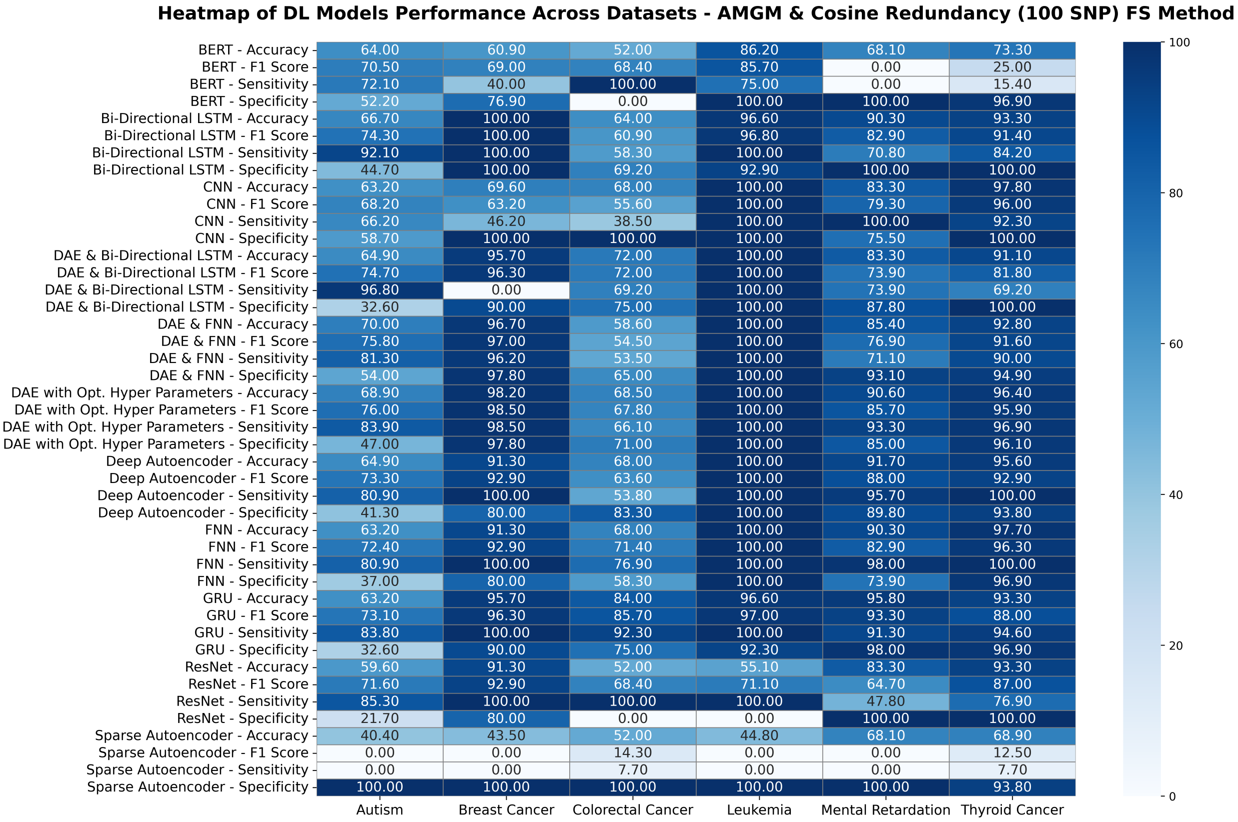

Figure 3a: Heatmap of Accuracy Metrics for Case/Control Datasets and AMGM with Cosine Similarity FS Method (100 SNPs)

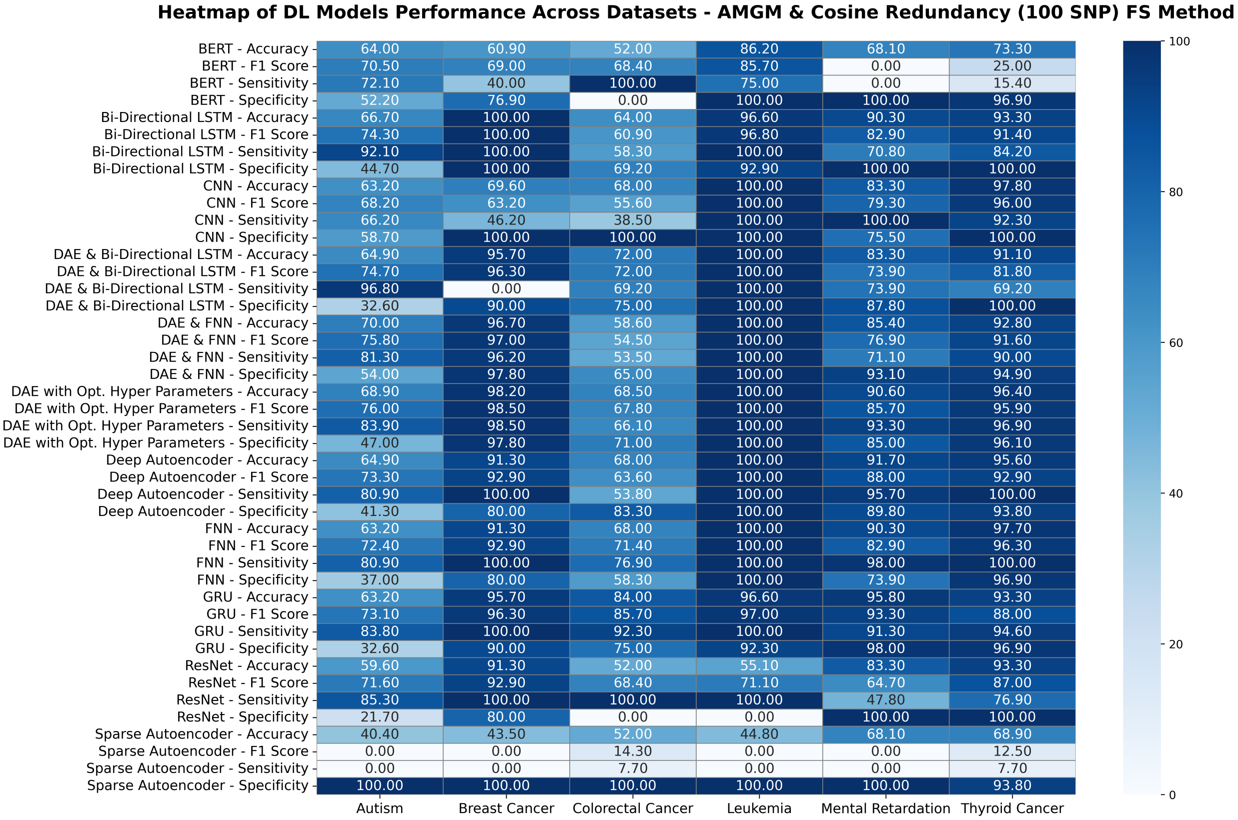

Figure 4a: Heatmap of Accuracy Metrics for Case/Control Datasets with L1 FS Method (100 SNPs)

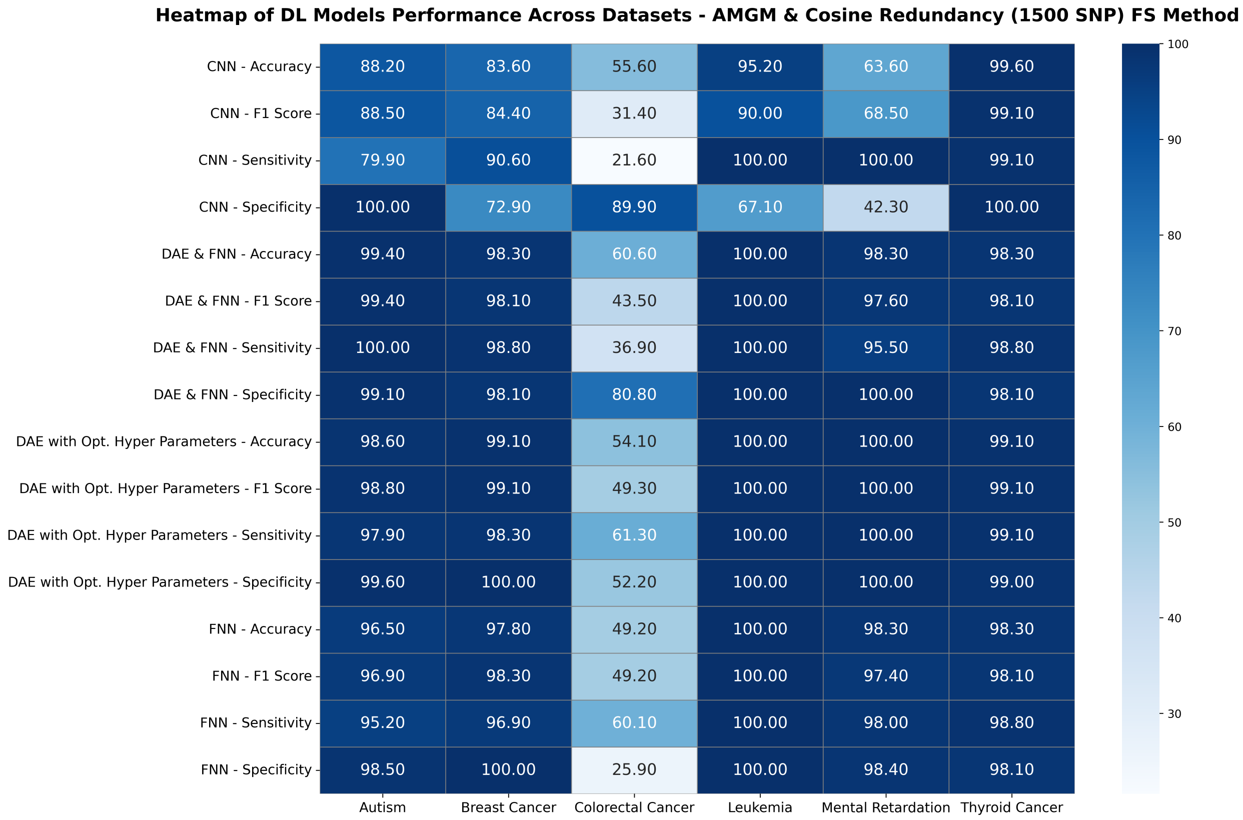

Figure 5a: Heatmap of Accuracy Metrics for Case/Control Datasets with Cosine Similarity FS Method (1500 SNPs)

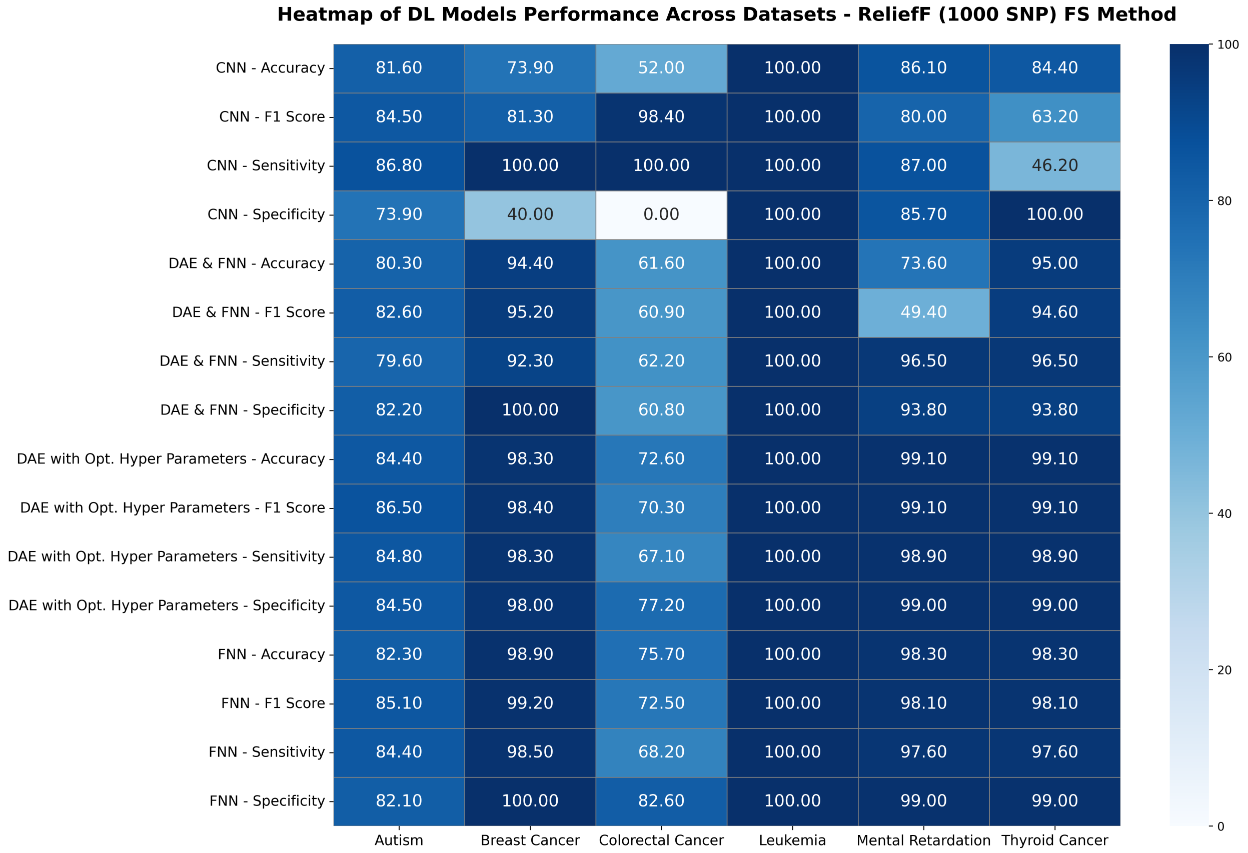

Figure 6a: Heatmap of Accuracy Metrics for Case/Control Datasets with ReliefF FS Method

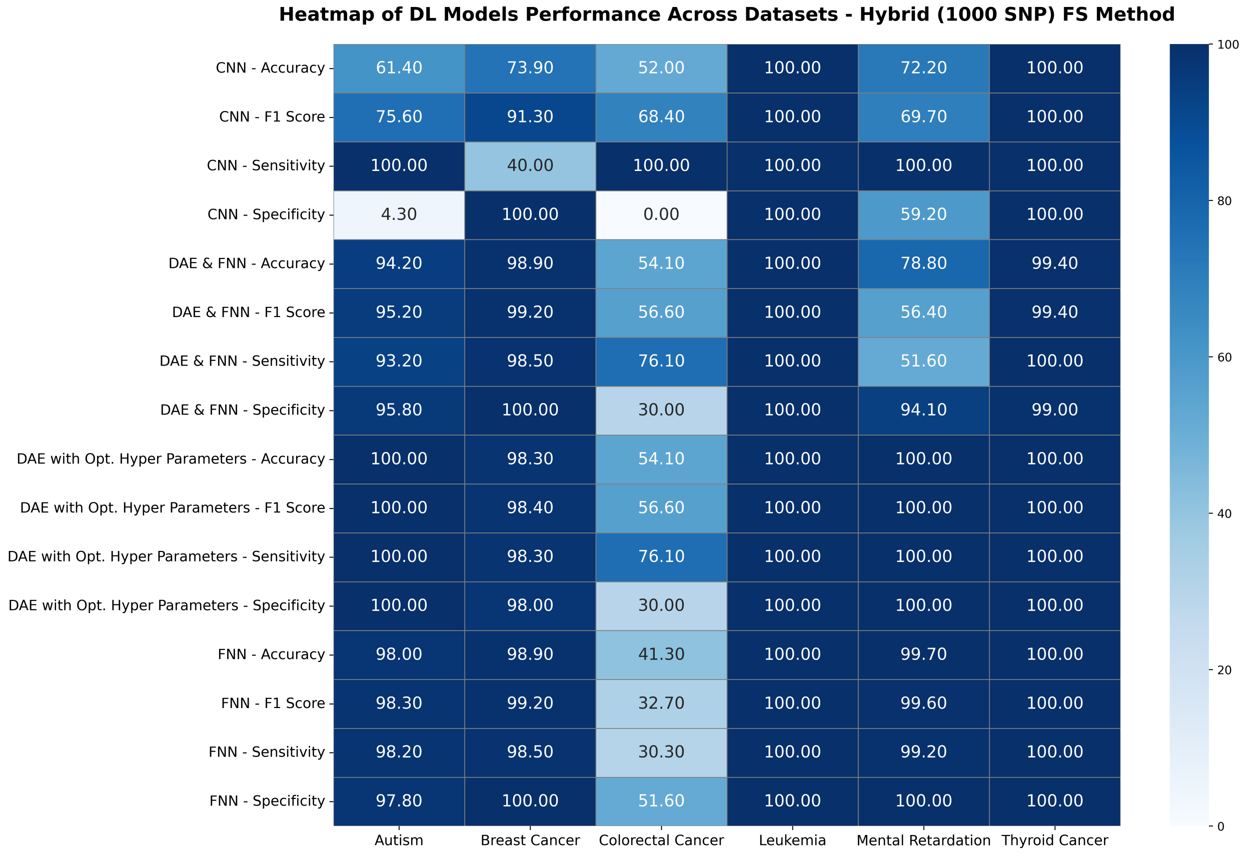

Figure 7a: Heatmap of Accuracy Metrics for Case/Control Datasets with Hybrid FS Method

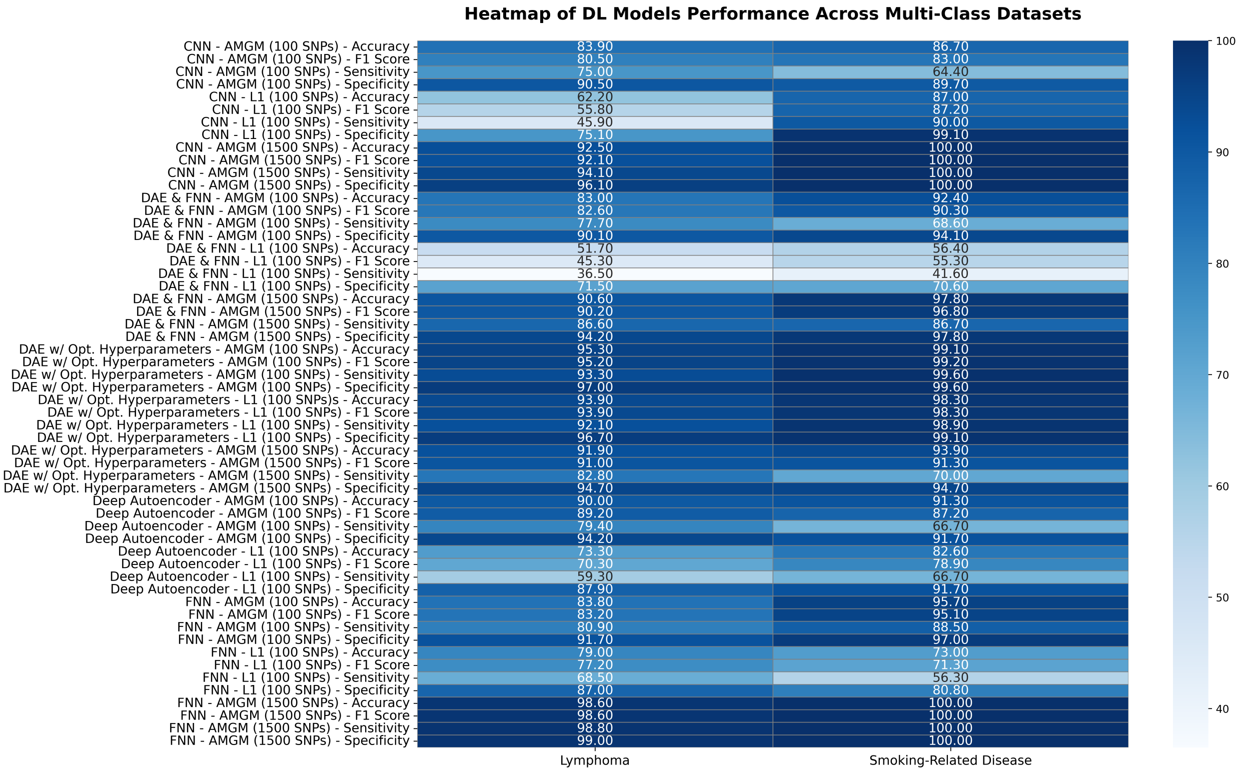

Figure 8a: Heatmap of Accuracy Metrics for Multi-Class Datasets for First 3 FS Methods

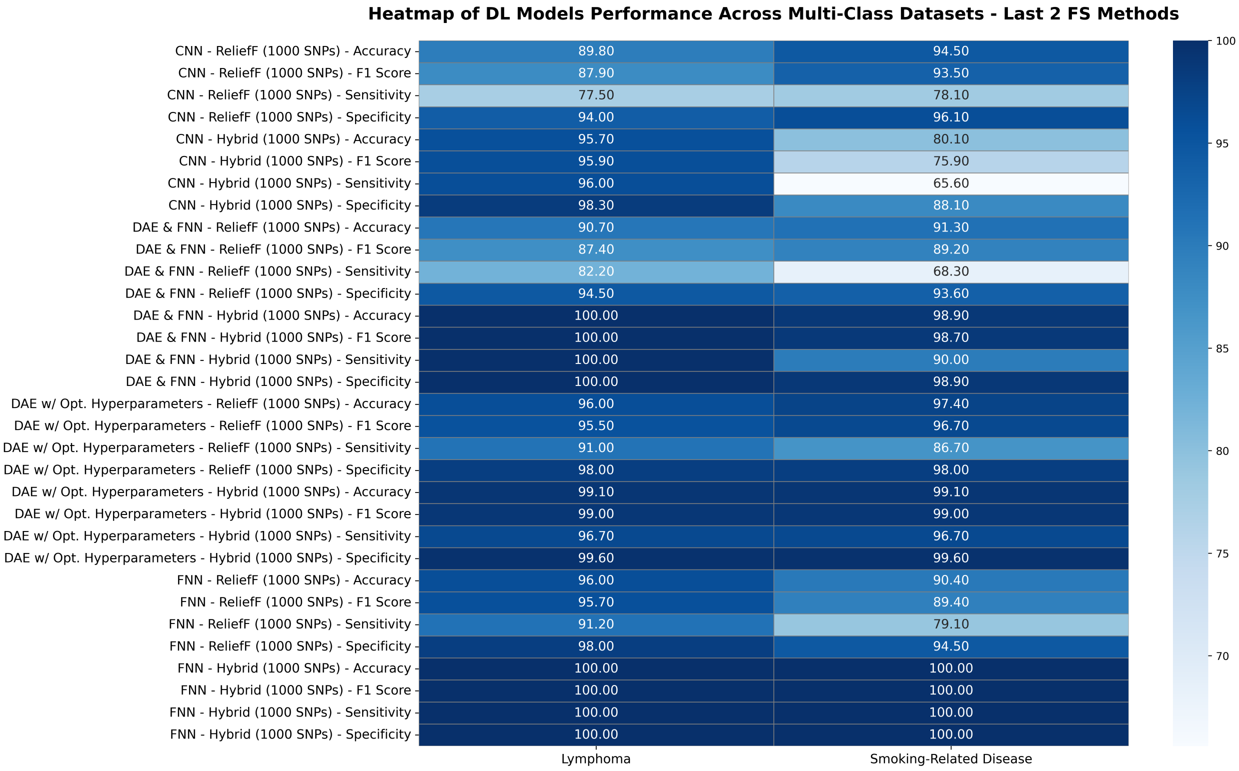

Figure 9a: Heatmap of Multi-Class Datasets for Last 2 FS Methods

Table 4a: Two Top-Performing Models For Each Dataset

Dataset	Model	FS Method	Accuracy	F-1 Score	Specificity	Sensitivity
Autism	Deep Autoencoder & FNN	AMGM (1500 SNPs)	99.4%	99.4%	99.1%	100%
Autism	FNN	Hybrid FS	98.0%	98.3%	97.8%	98.2%
Breast Cancer	Deep Autoencoder	AMGM (1500 SNPs)	99.1%	99.1%	100%	98.3%
Breast Cancer	FNN	Hybrid FS or ReliefF	98.9%	99.2%	100%	98.5%
Colorectal Cancer	GRU	AMGM (100 SNPs)	84.0%	85.7%	75.0%	92.3%
Colorectal Cancer	FNN	ReliefF	75.7%	72.5%	82.6%	68.2%
Leukemia	Bi-Directional LSTM	AMGM (100 SNPs)	96.6%	96.8%	92.9%	100%
Leukemia	CNN	AMGM (1500 SNPs)	85.2%	90.0%	67.7%	100%
Mental Retardation	FNN	Hybrid FS	99.7%	99.6%	100%	99.2%
Mental Retardation	Deep Autoencoder & FNN	AMGM (1500 SNPs)	98.3%	97.6%	100%	95.5%
Thyroid Cancer	CNN	AMGM (1500 SNPs)	99.6%	99.1%	100%	99.1%
Thyroid Cancer	Deep Autoencoder & FNN	Hybrid FS	99.4%	99.4%	99.0%	100%
Lymphoma	FNN	AMGM (1500 SNPs)	98.6%	98.6%	99.0%	98.8%
Lymphoma	Deep Autoencoder	Hybrid FS	98.0%	97.9%	94.5%	95.3%
Smoking-Related Disease	Deep Autoencoder	AMGM (100 SNPs)	99.1%	99.2%	99.6%	99.6%
Smoking-Related Disease	Deep Autoencoder	Hybrid FS	99.1%	99.0%	99.6%	96.7%

Table 5a: Hyperparameters of Top-Performing Model for Each Dataset

Dataset	Model	FS Method	Epochs	Dropout Rate	Batch Size	Learning Rate	Neurons 1	Neurons 2
Autism	Deep Autoencoder & FNN	AMGM (1500 SNPs)	150	0.611	112	0.00015	128	128
Breast Cancer	Deep Autoencoder	AMGM (1500 SNPs)	50	0.412	96	0.059	96	48
Colorectal Cancer	GRU	AMGM (100 SNPs)	20	0.261	48	0.0082	64 (Units)	N/A
Leukemia	Bi-Directional LSTM	AMGM (100 SNPs)	30	0.211	32	0.0048	64 (Units)	N/A
Mental Retardation	FNN	Hybrid FS	140	0.113	96	0.0042	224 (Units)	N/A
Thyroid Cancer	CNN	AMGM (1500 SNPs)	40	0.397	32	0.0045	64 (Filter Size)	4 (Kernel Size)
Lymphoma	FNN	AMGM (1500 SNPs)	70	0.222	80	0.0085	128 (Units)	N/A
Smoking-Related Disease	Deep Autoencoder	AMGM (100 SNPs)	100	0.497	112	0.0074	160	96
